# Factors associated with ventilator-associated pneumonia and outcomes in mechanically ventilated patients with nontraumatic intracerebral hemorrhage: a real-world data analysis

**DOI:** 10.62675/2965-2774.20260309

**Published:** 2026-05-18

**Authors:** Andrea Loggini, Adnan I. Qureshi, Christos Lazaridis, Faddi G. Saleh Velez, Victor J. Del Brutto, Awni D. Shahait, Amber Schwertman, Antoni Torres Marti, Chiara Robba, Denise Battaglini

**Affiliations:** 1 Brain and Spine Institute Southern Illinois Healthcare Carbondale Illinois United States Brain and Spine Institute, Southern Illinois Healthcare - Carbondale, Illinois, United States.; 2 University of Missouri Zeenat Qureshi Stroke Institutes and Department of Neurology Columbia Missouri United States Zeenat Qureshi Stroke Institutes and Department of Neurology, University of Missouri - Columbia, Missouri, United States.; 3 University of Chicago Division of Neurocritical Care Department of Neurology Chicago Illinois United States Department of Neurology, Division of Neurocritical Care, Medical Center, University of Chicago - Chicago, Illinois, United States.; 4 University of Oklahoma Department of Neurology Brain Stimulation and Neurorehabilitation Laboratory Oklahoma City Oklahoma United States Brain Stimulation and Neurorehabilitation Laboratory, Department of Neurology. Health Sciences Center, University of Oklahoma - Oklahoma City, Oklahoma, United States.; 5 University of Miami Miller School of Medicine Department of Neurology Miami Forida United States Department of Neurology, Miller School of Medicine, University of Miami - Miami - Forida, United States.; 6 Southern Illinois Healthcare Department of Surgery Section of Trauma & Acute Care Surgery Carbondale Illinois United States Section of Trauma & Acute Care Surgery, Department of Surgery, Southern Illinois Healthcare - Carbondale, Illinois, United States.; 7 Southern Illinois University School of Medicine Carbondale Illinois United States School of Medicine, Southern Illinois University - Carbondale, Illinois, United States.; 8 University of Barcelona Institut D’Investigacions August Pi I Sunyer School of Medicine Barcelona Spain School of Medicine, Institut D’Investigacions August Pi I Sunyer, University of Barcelona - Barcelona, Spain.; 9 IRCCS Ospedale Policlinico San Martino Departmento of Anesthesia and Intensive Care Genova Italy Departmento of Anesthesia and Intensive Care, IRCCS Ospedale Policlinico San Martino - Genova, Italy.

**Keywords:** Cerebral hemorrhage, Ischemic stroke, Ventilator-associated pneumonia, Length of stay, Cost of hospitalization

## Abstract

**Objective:**

To investigate the association between ventilator-associated pneumonia and outcomes in mechanically ventilated nontraumatic intracerebral hemorrhage patients.

**Methods:**

Retrospective data analysis from the National Inpatient Sample database, including patients from 2008 to 2022, for adult hospitalized intracerebral hemorrhage patients on mechanical ventilation. Variables included age, sex, race, hospital location, comorbidities, indicators of intracerebral hemorrhage severity, and neurosurgical procedures. The cohort was divided into ventilator-associated pneumonia and non-ventilator-associated pneumonia groups. Propensity-score matching was applied to balance comorbidities and severity between the two groups. Binary logistic regression was used to analyze the predetermined outcomes. P value was set at 0.05.

**Results:**

Of 70,870 mechanically ventilated intracerebral hemorrhage patients, 3,183 (4.5%) developed ventilator-associated pneumonia. Intracerebral hemorrhage patients with ventilator-associated pneumonia were younger (59 [49 - 69] *versus* 65 [54 - 76]), more frequently male (62.3% *versus* 54.3%), and more frequently black (27.4*% versus* 21.2%), p < 0.001 for all. Ventilator-associated pneumonia patients had a higher rate of cerebral edema (50.7% *versus* 37%), brain compression (32.5% *versus* 27.3%), obstructive hydrocephalus (36.1% *versus* 24.7%), and neurosurgical procedures, both external ventricular drain (38.2% *versus* 20.2%) and hematoma evacuation (13.8% *versus* 8.6%), p < 0.001 for all. Time from presentation to intubation was longer in ventilator-associated pneumonia (days; 0 [0 - 3] *versus* 0 [0 - 1], p < 0.001). After 1:1 propensity-score matching, binary logistic regression revealed that ventilator-associated pneumonia remained independently associated with prolonged length of hospital stay (OR = 1.56, 95%CI 1.3 - 1.82; p < 0.01), higher hospitalization cost (OR = 1.47, 95%CI 1.26 - 1.7; p < 0.01) and higher odds of unfavorable discharge disposition (OR = 1.26, 95%CI 1.06 - 1.49; p = 0.01).

**Conclusion:**

Ventilator-associated pneumonia significantly complicates the care of mechanically ventilated intracerebral hemorrhage patients, increasing the healthcare resource utilization, prolonging length of stay, and being associated with worse short-term functional outcomes.

## INTRODUCTION

Nontraumatic intracerebral hemorrhage (ICH) represents one of the most devastating forms of stroke, accounting for 10 - 15% of all stroke cases, yet contributing disproportionately to high morbidity and mortality.^(1,2)^ Despite advances in neurocritical care and supportive strategies, outcomes remain poor, with in-hospital mortality rates exceeding 20% and significant long-term disability among survivors.^(3,4)^ A substantial proportion of ICH patients require intensive care unit (ICU) admission and invasive supportive measures, including mechanical ventilation, due to decreased level of consciousness, airway compromise, or associated medical complications.^(5,6)^

Among critically ill patients with ICH, ventilator-associated pneumonia (VAP) is a relevant complication. Ventilator-associated pneumonia is defined as pneumonia occurring more than 48 hours after endotracheal intubation; it is a well-characterized condition in general critical care, and it is associated with increased risk of systemic complications, prolonged intensive care unit (ICU) stay, and worse discharge outcomes.^(7–9)^ In the broader critical care literature, VAP has been extensively studied, with several preventive strategies and care management implemented to prevent and mitigate its effects.^(10)^ However, in patients with ICH, who often have a significantly depressed level of consciousness, limited spontaneous mobility, and impaired protective airway reflexes, the risk of VAP may be uniquely high, and its implications particularly unfavorable. The interplay between VAP and the pathophysiology of acute brain injury is complex: systemic infections can exacerbate neuroinflammation, disrupt cerebral autoregulation, and trigger secondary injury mechanisms that worsen neurological outcomes.^(11,12)^

This study aims to investigate the association between VAP and outcomes in mechanically ventilated nontraumatic ICH patients. Primary outcome included in-hospital metrics (length of stay, cost, discharge disposition). Secondary outcome included procedural interventions (gastrostomy and tracheostomy).

## METHODS

### Study design and data source

We conducted a retrospective cohort study using data from the National Inpatient Sample (NIS) database, covering January 2008 to December 2022. The NIS is the largest publicly available all-payer inpatient database in the United States and includes a 20% stratified sample of all hospital discharges, weighted to estimate national hospitalizations. Adult patients (age ≥ 18) admitted with a diagnosis of nontraumatic ICH (International Classification of Diseases [ICD]-9 codes: 431, 432; ICD-10 codes: I61x, I629) and who underwent mechanical ventilation were identified. Patients were further categorized based on the presence or absence of VAP (ICD-9 code: 99731; ICD-10 code: J95851). Patients with missing outcome data were excluded. This study was performed according to the Strengthening the Reporting of Observational Studies in Epidemiology (STROBE) statement guidelines for observational cohort studies and followed the Good Clinical Practice Guidelines (GCP) and the principles of the Declaration of Helsinki. Institutional Review Board approval and informed consent were not required for this study because the dataset was deidentified.

### Data selection

Demographic variables included age, sex, and race. Clinical variables included comorbidities (hypertension, diabetes, chronic kidney disease, obesity, chronic obstructive pulmonary disease [COPD]), anticoagulant use, smoking, and time from presentation to intubation. Markers of ICH severity were coma, cerebral edema, brain compression, and obstructive hydrocephalus. Neurosurgical interventions included external ventricular drain (EVD) or ventriculoperitoneal shunt placement and hematoma evacuation/decompression. Neurological complications included acute ischemic stroke, seizures; medical complications included deep vein thrombosis, pulmonary embolism, acute kidney injury, sepsis, and acute respiratory distress syndrome (ARDS). Outcomes included hospital procedures (gastrostomy, tracheostomy) and in-hospital outcomes (length of stay, hospitalization cost, discharge disposition). Variables were identified by ICD-9 and ICD-10 codes (Table 1S - Supplementary Material).

### Definitions

Ventilator-associated pneumonia was defined as pneumonia developing 48 hours or more after endotracheal intubation and mechanical ventilation, where the infection was not present before intubation (ICD-10 code from October 2015 to December 2022) and as pneumonia that arises in a patient receiving mechanical ventilation (ICD-9 code from January 2008 to September 2015). Non-VAP was defined as patients who received mechanical ventilation but did not have ICD-9 or ICD-10 codes for VAP. Prolonged length of stay (PLOS) was defined as length of stay exceeding the 75^th^ percentile of the entire cohort, and within each subgroup analyzed. The threshold for a higher hospitalization cost was set at the 75^th^ percentile of the entire cohort, and within each subgroup, it was analyzed. The cost of hospitalization was adjusted for inflation to 2022 based on publicly available Consumer Price Index (CPI) data. An unfavorable discharge disposition was defined as in-hospital mortality or discharge to a setting other than home or acute inpatient rehabilitation.

### Statistical analysis

Continuous variables were presented as medians with interquartile ranges (IQR), and categorical variables as frequencies and percentages. Between-group differences were assessed using Mann-Whitney U tests and Chi-squared tests. Propensity score matching (PSM) was applied to balance baseline demographics (age, sex, race), comorbidities (hypertension, diabetes mellitus, chronic kidney disease, COPD, obesity), use of anticoagulants, tobacco use, hospital location, ICH severity (coma, cerebral edema, brain compression, and obstructive hydrocephalus) and neurosurgical procedures (EVD and hematoma evacuation) between patients with and without VAP. A 1:1 nearest-neighbor greedy matching without replacement was performed, using a caliper of 0.1 standard deviations of the logit of the propensity score to derive matched pairs. Balance was verified by standardized mean differences (< 0.1 threshold), Love plots, and overlap plots. Logistic regression was used to assess the independent association of VAP with each predetermined outcome in the matched cohort. Effect modification by age and sex was assessed by including interaction terms between VAP and the stratifying variable (sex, age ≥ 65, or age ≥ 80) in the logistic regression models. Interaction p-values were derived from the interaction term's coefficient to test for statistically significant heterogeneity of effects across subgroups. To evaluate the robustness of significant associations to potential unmeasured confounding, E-values were calculated for each outcome using the point estimate and the lower bound of the 95% confidence interval. Significance was set at p < 0.05. All statistical analyses were computed using Statistical Package for Social Sciences (SPSS) Version 29.0.2.0 (IBM Corp., Armonk, NY).

## RESULTS

### Baseline characteristics

**Figure 1:**
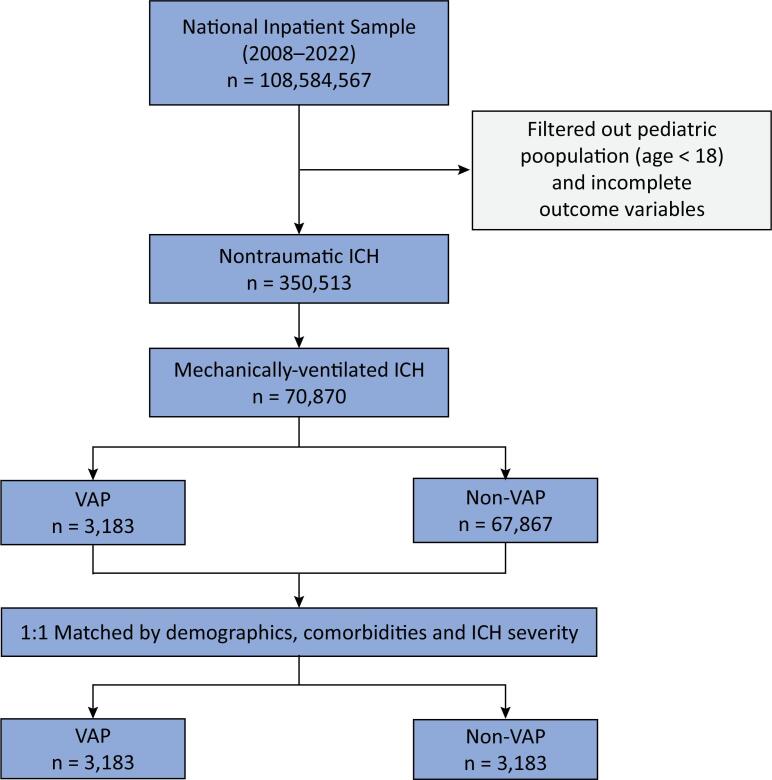
Study flowchart.

The study flowchart is depicted in figure 1. Of 70,870 mechanically ventilated ICH patients identified, 3,183 (4.5%) developed VAP. Ventilator-associated pneumonia patients were younger (59 [49 - 69] *versus* 65 [54 - 76], p < 0.001), more likely to be male (62.3% *versus* 54.3%) and black (27.4% *versus* 21.2%). They were more frequently treated in teaching hospitals (91.7% *versus* 78.5%). Ventilator-associated pneumonia patients had higher rates of cerebral edema (50.7% *versus* 37%), brain compression (32.5% *versus* 27.3%), and obstructive hydrocephalus (36.1% *versus* 24.7%). Neurosurgical procedures were more common among VAP patients, including EVD placement (38.2% *versus* 20.2%) and hematoma evacuation (13.8% *versus* 8.6%), p < 0.001. Time from presentation to intubation was longer in VAP, days (0 [0 - 3] *versus* 0 [0 - 1], p < 0.001). Ventilator-associated pneumonia patients had significantly higher rates of complications, including acute ischemic stroke (25.5% *versus* 19%), acute kidney injury (42.1% *versus* 27.9%), pulmonary embolism (6.4% *versus* 2.9%), sepsis (38.2% *versus* 17.5%), and septic shock (16.6% *versus* 8.1%). They also underwent gastrostomy (50.7% *versus* 19.6%) and tracheostomy (53.6% *versus* 13.7%) more frequently (p < 0.01 for both). Median length of stay was longer (26 [17 - 40] *versus* 8 [2 - 19] days), and hospitalization costs were significantly higher (452,178 [258,318 - 785,440] *versus* 158,227 [61,006 - 341,490] $) (Table 1).

ICH - intracerebral hemorrhage; VAP - ventilator - associated pneumonia.

**Table 1:** Baseline characteristics of the cohort and univariate analyses

Variables		Non-VAP (n = 67,687)	VAP (n = 3,183)	p value	Matched non-VAP (n = 3,183)	SMD
Median age (years)		65 [54 - 76]	59 [49 - 69]	< 0.01	60 [49 - 70]	-0.0206
Women		30,897 (45.7)	1,199 (37.7)	< 0.01	1,158 (36.4)	0.0266
Race				< 0.01		-0.0163
	White	36,673 (56.2)	1,355 (44.5)		1,385 (45.7)	
	Black	14,281 (21.2)	853 (27.4)		788 (25.2)	
	Hispanic	8,506 (12.1)	473 (14.4)		506 (15.5)	
	Asian	4,284 (5.5)	240 (6.5)		228 (6)	
	Others	3,943 (5)	262 (7.2)		276 (7.7)	
Hospital location						-0.0285
	Teaching	53,168 (78.5)	2,919 (91.7)	< 0.01	2,944 (92.5)	
	Nonteaching	14,519 (21.5)	264 (8.3)	< 0.01	239 (7.5)	
Comorbidities						
	Hypertension	50,245 (74.2)	2,118 (66.5)	< 0.01	2,131 (66.9)	-0.0087
	Diabetes mellitus	19,460 (28.7)	840 (26.4)	0.37	844 (26.5)	-0.0029
	Chronic kidney disease	11,316 (16.7)	485 (15.2)	0.03	498 (15.6)	-0.0114
	COPD	6,756 (10)	250 (7.9)	< 0.01	233 (7.3)	0.0199
	Obesity	7,553 (11.2)	420 (13.2)	< 0.01	393 (12.3)	0.0251
	Use of anticoagulant	6,436 (9.5)	145 (4.6)	< 0.01	106 (3.3)	0.0588
	Tobacco use	15,775 (23.3)	554 (17.4)	< 0.01	544 (17.1)	0.0083
ICH severity						
	Coma	8,414 (12.4)	346 (10.9)	< 0.01	279 (8.8)	0.0676
	Cerebral edema	25,059 (37)	1,614 (50.7)	< 0.01	1,631 (51.2)	-0.0107
	Brain compression	18,493 (27.3)	1,035 (32.5)	< 0.01	990 (31.1)	0.0302
	Obstructive hydrocephalus	16,705 (24.7)	1,150 (36.1)	< 0.01	1,078 (33.9)	0.0471
Neurosurgical procedures						
	EVD/VPS	13,698 (20.2)	1,217 (38.2)	< 0.01	1,143 (35.9)	0.0478
	Hematoma removal/decompression	5,793 (8.6)	438 (13.8)	< 0.01	403 (12.7)	0.0319
In-hospital complications						
	Deep vein thrombosis	3,368 (5)	370 (11.6)	< 0.01	209 (6.6)	-
	Pulmonary embolism	1,934 (2.9)	205 (6.4)	< 0.01	101 (3.2)	-
	Acute kidney injury	18,872 (27.9)	1,339 (42.1)	< 0.01	985 (30.9)	-
	Sepsis	11,843 (17.5)	1,217 (38.2)	< 0.01	641 (20.1)	-
	Septic shock	5,462 (8.1)	529 (16.6)	< 0.01	285 (9)	-
	ARDS	792 (1.2)	58 (1.8)	< 0.01	38 (1.2)	-
	Acute ischemic stroke	12,872 (19)	813 (25.5)	< 0.01	597 (18.8)	-
	Seizures	7,942 (11.7)	400 (12.6)	0.73	383 (12)	-
Procedures						
	Time from presentation to intubation (days)	0 [0 - 1]	0 [0 - 3]	< 0.01	0 [0 - 2]	-
	Gastrostomy	13,249 (19.6)	1,614 (50.7)	< 0.01	787 (24.7)	-
	Tracheostomy	9,296 (13.7)	1,705 (53.6)	< 0.01	585 (18.4)	-
Outcome measures						
	Length of hospital stay (days)	8 [3 - 19]	26 [17 - 40]	< 0.01	12 [4 - 23]	-
	Cost of hospitalization ($)	158,227 [61,006 - 341,490]	452,178 [258,318 - 785,440]	< 0.01	220,077 [94,726 - 429,395]	-
	Discharge to home or acute rehab	6,335 (9.3)	265 (8.3)	0.06	327 (10.3)	-
	Unfavorable discharge disposition and mortality	61,352 (90.7)	2,918 (91.7)	0.06	2854 (89.7)	-

VAP - ventilator - associated pneumonia; SMD - standardized mean difference; COPD - chronic obstructive pulmonary disease; ICH - intracerebral hemorrhage; EVD - external ventricular drain; VPS - ventriculoperitoneal shunt; ARDS - acute respiratory distress syndrome. Results expressed as median (interquartile range) or n (%).

### Adjusted outcomes in the propensity-matched cohort

After applying 1:1 propensity score matching to control for confounding factors (Table 1, Figures 1 - 3 [Supplementary Material]), logistic regression analysis revealed that VAP remained independently associated with a significantly increased likelihood of undergoing gastrostomy (OR 1.32, 95%CI 1.15 - 1.53) and tracheostomy (OR 3.26, 95%CI 2.81 - 3.78). Additionally, VAP was associated with higher odds of prolonged length of stay (odds ratio [OR] 1.56, 95% confidence interval [95%CI] 1.33 - 1.82), higher hospitalization costs (OR 1.47, 95%CI 1.26 - 1.7), and greater odds of an unfavorable discharge disposition (OR 1.26, 95%CI 1.06 - 1.49) (Table 2).

**Table 2:** Binary logistic regression analysis of predetermined in-hospital procedures and outcomes in ventilator-associated pneumonia

		OR	95%CI	p value
Procedures				
	Gastrostomy	1.32	1.15 - 1.53	< 0.01
	Tracheostomy	3.26	2.81 - 3.78	< 0.01
Hospital outcomes				
	Length of hospital stay > 32 days	1.56	1.33 - 1.82	< 0.01
	Cost of hospitalization > $614,997	1.47	1.26 - 1.70	< 0.01
	Unfavorable discharge disposition	1.26	1.06 - 1.49	0.01

OR - odds ratio; 95%CI - 95% confidence interval. 1:1 propensity score matching cohort.

### Stratified analyses by age and sex

Age- and sex-stratified analyses demonstrated heterogeneity in outcomes (Table 3). Among patients ≥ 80, VAP was significantly associated with prolonged LOS (OR: 2.85, 95%CI 1.60 - 5.07; interaction p value: 0.01), but not with unfavorable discharge. Both male and female patients had increased odds of sepsis and tracheostomy, with women showing a stronger association with prolonged LOS (OR 1.87, 95%CI 1.44 - 2.44; interaction p value 0.02) without increased odds of unfavorable outcomes (Table 2S - Supplementary Material).

**Table 3:** Binary logistic regression stratified analyses of predetermined in-hospital procedures and outcomes in ventilator-associated pneumonia with relative interaction p value

			OR	95%CI	p value	Interaction p value
Male						
	Procedures					
		Gastrostomy	1.23	1.03 - 1.48	0.02	-
		Tracheostomy	3.33	2.76 - 4.02	< 0.01	-
	Hospital outcomes					
		Length of hospital stay > 32 days	1.41	1.16 - 1.72	< 0.01	-
		Cost of hospitalization > $ 614,997	1.46	1.21 - 1.75	< 0.01	-
		Unfavorable discharge disposition	1.26	1.01 - 1.56	0.04	-
Female						
	Procedures					
		Gastrostomy	1.51	1.2 - 1.91	< 0.01	0.12
		Tracheostomy	3.11	2.45 - 3.96	< 0.01	0.41
	Hospital outcomes					
		Length of hospital stay > 32 days	1.87	1.44 - 2.44	< 0.01	0.02
		Cost of hospitalization > $ 614,997	1.49	1.15 - 1.91	0.01	0.31
		Unfavorable discharge disposition	1.27	0.96 - 1.67	0.09	0.97
Age ≥ 65						
	Procedures					
		Gastrostomy	1.30	1.03 - 1.65	0.03	0.11
		Tracheostomy	3.17	2.47 - 4.07	< 0.01	< 0.01
	Hospital outcomes					
		Length of hospital stay > 29 days	2.04	1.56 - 2.66	< 0.01	< 0.01
		Cost of hospitalization > $ 549,337	1.58	1.23 - 2.04	< 0.01	< 0.01
		Unfavorable discharge disposition	1.01	0.68 - 1.5	0.97	< 0.01
Age ≥ 80						
	Procedures					
		Gastrostomy	1.04	0.58 - 1.87	0.89	0.01
		Tracheostomy	4.27	2.18 - 8.34	< 0.01	< 0.01
	Hospital outcomes					
		Length of hospital stay > 21 days	2.85	1.6 - 5.07	< 0.01	< 0.01
		Cost of hospitalization > $ 371,262	1.43	0.82 - 2.49	0.21	< 0.01
		Unfavorable discharge disposition	0.97	0.36 - 2.65	0.96	0.01

OR - odds ratio; 95%CI - 95% confidence interval. 1:1 propensity score matching cohort.

## DISCUSSION

In this nationally representative cohort of mechanically ventilated patients with nontraumatic ICH, VAP emerged as an independent factor associated with worse clinical outcomes, greater procedural intervention, and higher healthcare resource utilization.

These findings reinforce the concept that VAP represents a marker of more complex and prolonged critical illness in patients with ICH.^(13,14)^ While patients who develop VAP may be inherently more critically ill, the persistence of adverse associations after score matching and adjustment suggests a substantial and independent contribution of VAP to the development of morbidity in this patient population.^(15)^

The first observation pertains to the incidence of VAP in our cohort (4.5%), which is lower than the 10 - 40%.^(14,16–18)^ range reported in the literature. It is important to note that our study is based on real-world data, outside a controlled observational environment. It includes non-teaching hospitals, which introduce variability in mechanical ventilation use among ICH patients. Additionally, the population examined excludes a broader group of traumatic brain injury (traumatic ICH), in which critical care is more complex and systemic complications are more frequent compared to nontraumatic ICH. A selection bias may also have occurred, including patients who were electively intubated for neurosurgical procedures (placement of an EVD), patients who died before developing VAP, or patients managed at centers with strong adherence to ventilator bundles in the United States.^(19–21)^ These factors may have contributed to the lower observed VAP rate in our cohort compared to prior reports.

While the methodology used for case ascertainment is likely highly specific, it may lack sensitivity, potentially underestimating the true incidence of VAP. This limitation is consistent with several validation studies that report low sensitivity but high specificity when using ICD-9 and ICD-10 codes to identify VAP.^(22,23)^ Administrative coding systems often fail to capture all true cases due to documentation and coding inconsistencies.^(24)^ Consequently, while prognostic outcomes linked to VAP may remain valid due to the high specificity of the case definition, the actual burden of disease is likely underestimated.

The relationship between VAP and ischemic stroke may reflect systemic inflammatory processes and cerebral hypoperfusion triggered by sepsis, which can exacerbate ischemic injury in a brain already vulnerable from hemorrhage and elevated intracranial pressure. Similarly, the association with acute kidney injury and pulmonary embolism may reflect the systemic consequences of infection and immobility in these critically ill patients.^(25)^

Time from presentation to intubation was significantly longer in patients who developed VAP. This finding may reflect several possible pathophysiological and clinical dynamics. Delayed intubation in the context of ICH could result from a more conservative initial management approach or clinical uncertainty about the trajectory of neurological deterioration. In some cases, neurointensivists may attempt to avoid invasive ventilation to better track the patients’ neurological status, particularly in patients with borderline mental status or fluctuating airway protection. However, prolonged periods of reduced consciousness without definitive airway control may increase the risk of aspiration, a well-established precursor to VAP.^(7,10)^ Notably, the time delay from presentation to intubation may also serve as a proxy for the overall efficiency and readiness of critical care delivery, with longer intervals reflecting potential systemic delays or differences in provider thresholds for escalating care. In contrast, earlier intubation may lead to more rapid implementation of ventilator bundles and earlier ICU admission, potentially mitigating infection risk. This observation supports the hypothesis that timing of intubation may not simply be a marker of illness severity, but also an actionable factor influencing VAP risk.

The striking increase in the likelihood of tracheostomy and gastrostomy among patients with VAP underscores the broader implications of nosocomial infections in neurocritical care. Prolonged ventilatory support often requires tracheostomy for airway protection and secretion management in mechanically ventilated ICH patients. At the same time, gastrostomy may be needed for safe and sustained enteral nutrition in patients with impaired consciousness or dysphagia. These procedures carry their own risks and are associated with long-term morbidity, caregiver burden, and increased resource demands.^(26)^

Our stratified analyses reveal interesting differences both by age and sex. Younger patients exhibited the highest odds of developing pulmonary embolism and sepsis and were also more likely to undergo tracheostomy and experience poor functional outcomes. This suggests that younger patients with ICH who develop VAP may follow a particularly severe clinical trajectory, possibly due to the selection of more aggressive treatment strategies and prolonged attempts at recovery. Conversely, among patients ≥ 80, VAP was significantly associated with medical complications and resource use, though not with discharge outcomes, possibly reflecting decisions to limit aggressive interventions.

Nuanced sex differences also emerged in our analysis. Women with VAP were more likely than men to require prolonged hospitalization and gastrostomy, whereas men exhibited higher odds of tracheostomy. According to prior research, VAP is an independent predictor of mortality in women, particularly when developed within seven days of admission.^(27)^ These differences may reflect sex-related variation in recovery trajectories or provider decision-making. Such disparities warrant further investigation to ensure equitable care delivery across demographic groups.^(15)^

Our findings contribute to a growing body of literature emphasizing the importance of infection prevention in neurocritical care settings. While protocols to reduce VAP, such as elevation of the head of the bed, oral hygiene, subglottic suctioning, and early mobilization, are standard in many ICUs, their implementation may be inconsistent in the context of acute brain injury.^(20,21)^ Sedation protocols, neuromonitoring, and concerns over elevated intracranial pressure may limit opportunities for early weaning or spontaneous breathing trials, prolonging intubation and predisposing patients to pulmonary infections. These challenges emphasize the need for multidisciplinary approaches that balance neuroprotection with infection control.

Finally, from a systems perspective, the significantly higher costs associated with VAP in ICH patients, nearly threefold in our study, highlight the broader implications for hospital resource planning, bundled payments, and quality benchmarks. Reducing the incidence of VAP may not only improve patient outcomes but also increase hospital efficiency and reduce financial burden.

Future research should aim to validate these findings in prospective cohorts and to examine the impact of targeted preventive interventions for VAP in the mechanically ventilated ICH population. Studies accounting for clinical details such as hematoma volume, imaging findings, and longitudinal follow-up are needed to better characterize the trajectory of recovery and long-term outcomes in ICH patients with VAP.^(7,28,29)^

### Limitations

This study is limited by its retrospective design and reliance on administrative coding, which may introduce misclassification bias. The timing of VAP onset relative to outcomes cannot be established, limiting causal inference. Despite propensity score matching to balance baseline demographics, comorbidities, ICH severity markers, and neurosurgical procedures, residual confounding is likely to persist in this analysis. The NIS lacks several important clinical variables, such as ventilator duration, treatment intensity for intracranial hypertension, neurological examination scores, and imaging findings, that may influence both the risk of VAP and clinical outcomes. Moreover, many of the observed associations are confounded by time-varying factors, as the occurrence of VAP is closely related to the duration of mechanical ventilation and critical care management strategies. As such, the findings of this study should be interpreted as associations rather than causal relationships.

Furthermore, the database lacks data on VAP timing; therefore, it is not possible to distinguish between early and late VAP or to sub-analyze them. The database lacks information on institutional ventilator management practices, such as ventilator bundles, daily sedation interruption, and standardized weaning protocols, which have been shown in clinical trials to influence ventilator duration and VAP rates. Functional outcomes beyond discharge were not available.

## CONCLUSION

Ventilator-associated pneumonia significantly complicates the hospital course of patients with mechanically ventilated intracerebral hemorrhage, being associated with a higher rate of procedural interventions, prolonged length of stay, higher costs, and worse short-term functional outcomes. These findings highlight the importance of infection prevention, early detection of ventilator-associated pneumonia, and tailored intensive care unit care management in this high-risk neurocritical care population.

## Data Availability

The data of this study are available from the Healthcare Cost and Utilization Project (HCUP) National Inpatient Sample (NIS). These data were used under license for the current study and are not publicly available. Data are available from HCUP upon completion of the required Data Use Agreement.
