# Supplementary material for: Factors associated with ventilator-associated pneumonia and outcomes in mechanically ventilated patients with nontraumatic intracerebral hemorrhage: a real-world data analysis
Source: Crit Care Sci. 2026 May 18;38:e20260309. doi: 10.62675/2965-2774.20260309 (PMC13399244; doi:10.62675/2965-2774.20260309)
Supplement: Supplementary Material [file 2965-2774-ccsci-38-e20260309-suppl01.pdf]

Critical Care Science
https://doi.org/10.62675/2965-2774.20260309

SUPPLEMENTARY MATERIAL

Factors associated with ventilator-associated pneumonia and outcomes in mechanically ventilated patients with nontraumatic intracerebral hemorrhage: a real-world data analysis

Andrea Loggini[1], Adnan I. Qureshi[2], Christos Lazaridis[3], Faddi G. Saleh Velez[4], Victor J. Del Brutto[5], Awni D. Shahait[6], Amber Schwertman[7], Antoni Torres Marti[8], Chiara Robba[9], Denise Battaglini[9]

Table 1S - ICD - 9 and ICD - 10 codes for variables identification

Variable	ICD - 9 codes	ICD - 10 codes
Nontraumatic intracerebral hemorrhage	431, 432	I61x, I629
Hypertension	401, 4010, 4011, 4019, 40200, 40201, 40210, 40211, 40290, 40291, 40300, 40301, 40310, 40311, 40390, 40391, 40400, 40401, 40402, 40403, 40410, 40411, 40412, 40413, 40490, 40491, 40492, 40493, 40501, 40509, 40511, 40519, 40591, 40599	I10, I110, I110, I120, I129, I1311, I132, I150, I151, I152, I158, I159
Diabetes mellitus	250xx	E08xx, E10xx, E11xx, E13xx
Chronic kidney disease	5851, 5852, 5853, 5854, 5855, 5856, 5859, 586	N18xx
Obesity	2780, 27801, 27802, 27803	E6601, E6609, E662, E663, E668, E669
Chronic obstructive pulmonary disease	49120, 49121, 49122, 4920, 4928, 496	J440, J441, J448, J449
Intubation	9604, 9605	0BH17EZ, 0BH18EZ
Ventilator - associated pneumonia	99731	J95851
Tracheostomy	311, 3121, 3129	0B11x
Gastrostomy	430, 431, 4311, 4319, 432	0DH60UZ, 0DH63UZ, 0DH64UZ, 0DH67UZ, 0DH68UZ
Acute respiratory distress syndrome	5185, 51882	J80x
Sepsis	038x, 99592, 78552	A40x, A41x, R6520, R6521
Septic shock	78552	R6521
Deep vein thrombosis	45183, 45189, 41519, 45340, 45341, 45342, 4538, 45381, 45382, 45383, 45384, 45385, 45386, 45387, 45388, 45389, 4539	I824xx
Pulmonary embolism	4150, 4151, 41511, 41512, 41513, 41519	I2601, I2609, I2692, I2693, I2694, I2699
Use of anticoagulants	V5861	Z7901
EVD/VPS	022, 0221, 0222, 0231, 0232, 0233, 0234, 0235, 0239	009600Z, 00960ZX, 00960ZZ, 009630Z, 00963ZX, 00963ZZ, 009640Z, 00964ZX, 00964ZZ
Clot evacuation/decompression	0124, 0125, 0132, 0139, 0141, 0142, 0151, 0152, 0153, 0159, 0207	0097xxx, 0098xxx, 0099xxx, 009Axxx, 009Bxxx, 009Cxxx, 009Dxxx, 0NBxxxx, 0NSxxxx, 0NRxxxx, 00B0xxx

EVD - external ventricular drain; VPS - ventriculoperitoneal shunt.

This is an open access article under the CC BY license (https://creativecommons.org/licenses/by/4.0/).

Table 2S - E-values for subgroup analyses

	E-values (point)	E-values (lower CI)
Overall		
Procedures		
Gastrostomy	2	1.6
Tracheostomy	6	5.1
Hospital outcomes		
Length of hospital stay > 32 days	2.5	2
Cost of hospitalization > $614,997	2.3	1.8
Unfavorable discharge disposition	1.8	1.3
Male		
Procedures		
Gastrostomy	1.8	1.2
Tracheostomy	6.1	5
Hospital outcomes		
Length of hospital stay > 32 days	2.2	1.6
Cost of hospitalization > $614,997	2.3	1.7
Unfavorable discharge disposition	1.8	1.1
Female		
Procedures		
Gastrostomy	2.4	1.7
Tracheostomy	5.7	4.3
Hospital outcomes		
Length of hospital stay > 32 days	3.2	2.2
Cost of hospitalization > $614,997	2.3	1.6
Unfavorable discharge disposition	1.8	1
Age ≥ 65		
Procedures		
Gastrostomy	1.9	1.2
Tracheostomy	5.8	4.4
Hospital Outcomes		
Length of hospital stay > 29 days	3.5	2.5
Cost of hospitalization > $549,337	2.5	1.8
Unfavorable discharge disposition	1.1	1
Age ≥ 80		
Procedures		
Gastrostomy	1.3	1
Tracheostomy	8	3.8
Hospital Outcomes		
Length of hospital stay > 21 days	5.1	2.6
Cost of hospitalization > $371,262	2.2	1
Unfavorable discharge disposition	1.2	1

CI - confidence interval.

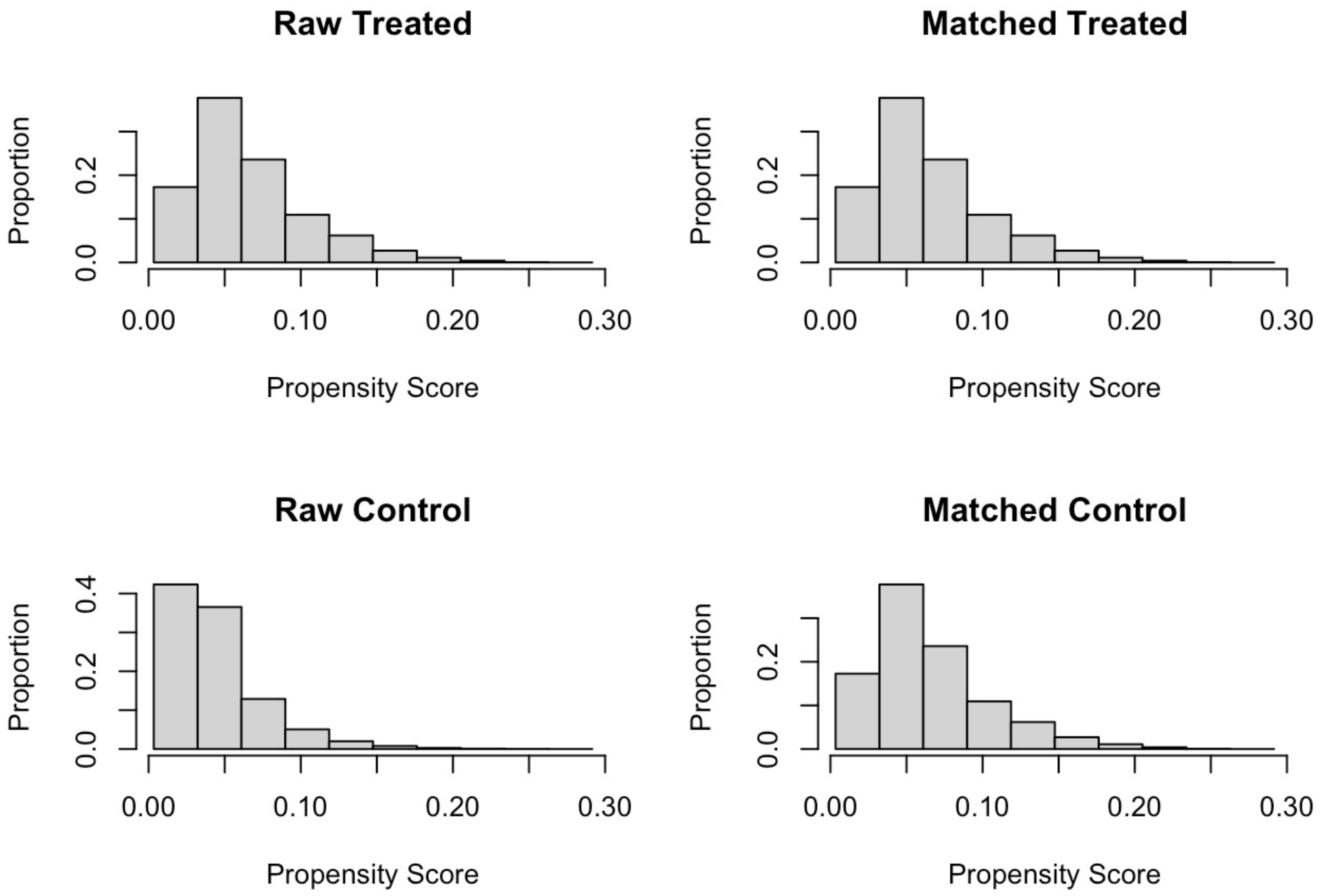

Figure 1S - Proportion of propensity scores between unmatched and matched groups.

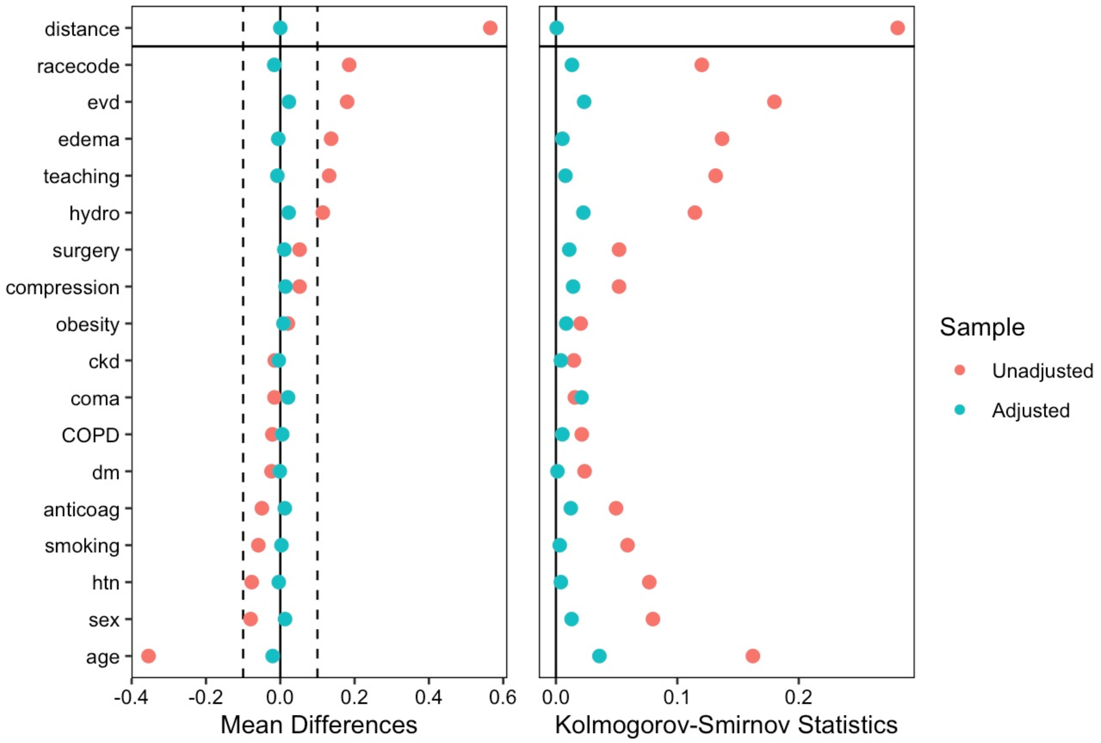

Figure 2S - Covariate balance.

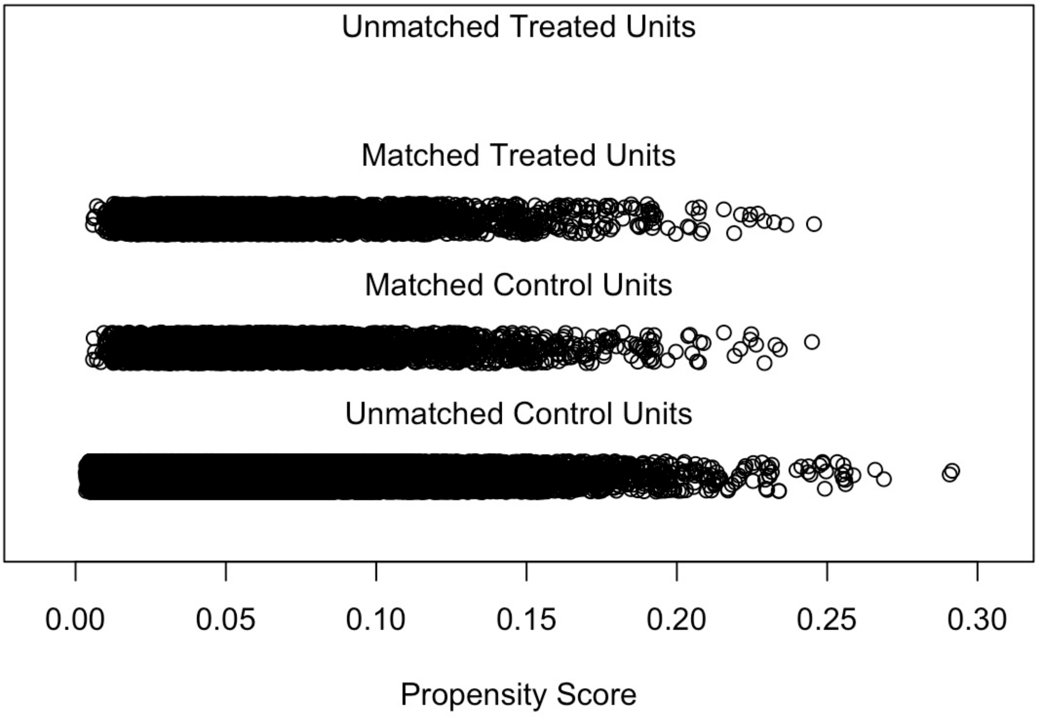

Figure 3S - Distribution of propensity scores.
